# Sexual harassment as experienced by nurses from selected healthcare facilities in Ghana

**DOI:** 10.1186/s12912-023-01228-6

**Published:** 2023-04-12

**Authors:** Aliu Mohammed, Edward Wilson Ansah, Daniel Apaak

**Affiliations:** Department of Health, Physical Education and Recreation, University of Cape Coast, Cape Coast, Ghana

**Keywords:** Sexual harassment, Nurses, Healthcare, Policy, Ghana

## Abstract

**Background:**

Workplace Sexual Harassment (WSH) remains a major occupational health hazard to many nurses globally. Despite the negative impact of WSH on health and safety of nurses, there are limited studies exploring sexual harassment experiences of nurses in the line of duty in Low- and middle-income countries (LMICs) including Ghana.

**Aim:**

This study aimed at exploring the lived sexual harassment experiences among nurses working in healthcare facilities in the Central Region of Ghana.

**Method:**

This study used a qualitative interpretive phenomenological approach (IPA). Data from 24 participants, 13 participants’ written descriptive narratives of sexual harassment experiences (via online) and 11 telephone interviews were analysed concurrently using the IPA.

**Results:**

Participants experienced verbal, non-verbal and physical forms of sexual harassment, often perpetrated by physicians, colleague workers, and patients. Most victims reacted passively to the acts sexual harassment and cases are often not reported despite the negative impact on victims’ health. While some participants dealt with sexual harassment by accepting the behaviour as an inevitable part of their job, others either quit or intend to quit the job to avoid the harassers. Most participants are unaware of any workplace measure such as policy that addresses sexual harassment in the health sector in Ghana.

**Conclusion:**

This study highlights the problems of sexual harassment against nurses in the healthcare sector in Ghana, and calls for urgent development of measures such as a sexual harassment policy to prevent occurrence and promote effective resolution of sexual harassment within the healthcare sector in the country. Sexual harassment against nurses could be hampering quality healthcare delivery in the region.

## Background

Sexual harassment remains a major problem in workplaces due to its significant negative impact on employees’ health, safety, productivity and organizational image [1, 2]. Available evidence suggests that workplace sexual harassment (WSH) affects victims’ psychological and physical health [3, 4] and may result in high levels of depressive symptoms [5], post-traumatic stress disorders (PTSD) [6] anxiety, fear [7], hypertension [8], and insomnia [9]. Workplace sexual harassment also contributes to productivity losses through absenteeism, presenteeism, job withdrawal, and attrition [3]. In Australia, for instance, the total economic cost of WSH in 2018 was estimated at 3.5 billion dollars, of which productivity cost (absenteeism, presenteeism, attrition and loss of manager time) accounted for 2.6 billion dollars while other costs (healthcare, legal fees, investigations, deadweight losses and justice system) was 0.9 billion dollars [10]. Besides, sexual harassment remains one of the most common workplace behaviours that often leads to attrition of nurses [11] and thereby contributes to nurse shortage and its negative ramifications on quality healthcare delivery.

Workplace sexual harassment refers to unwanted sexual conduct of any form (verbal, non-verbal or physical) which affects a person’s dignity and creates a hostile, intimidating, and offensive working environment to the victim [12]. Generally, WSH is classified into the “*quid pro quo*” and the “hostile working environment” [12]. The “*quid pro quo*” occurs when a worker’s willingness or unwillingness to engage in a sexual conduct is used as a precondition for some form of job benefits such as promotion, pay rise, continued employment or otherwise [11]. On the other hand, hostile working environment involves the creation of an offensive and unwelcome environment through the use of sexually suggestive comments, innuendos, insults or the display of sexually explicit materials [12].

Although most workers are exposed to sexual harassment [13], the prevalence of WSH often varies based on geographic location, type of organization studied, and approach used [3]. Healthcare workers, especially nurses, are known to have one of the highest risks for sexual harassment [14, 15], largely due to the close working relationship between nurses and patients or other workers [16]. It is estimated that one in every four nurses worldwide experience sexual harassment in the line of duty [17]. For instance, a recent systematic review of quantitative studies on sexual harassment against nurses found that 10 to 87.3% of female nurses worldwide have experienced sexual harassment at work [18], indicating a wide disparity across countries. In China, for instance, a meta-analytic study reported a prevalence of 7.5% of sexual harassment of nurses [19], whereas in Australia, the figure was 60% for female nurses and 34% for male nurses [20]. In Turkey, it was 37% [21] and 12.2% in Ghana [22]. Meanwhile, most victims, including nurses do not report or make formal complaint due to fear of victim blaming, or inaction from management [14, 23]. This often contributes to underestimation of sexual harassment cases in nursing [24] and perhaps, affect implementation of the needed policies to address the canker.

Although the issue of WSH has been widely investigated in high-income countries and various measures implemented towards minimizing the phenomenon [25], very limited studies have been conducted in LMICs. Meanwhile, there is relatively high prevalence of sexual harassment against nurses in many LMICs [26, 27] including Ghana [22], a situation that calls for urgent strategies to address the phenomenon. Besides, there are very limited interventions to address WSH in Ghana like in many other LMICs [25]. The few studies available focused on prevalence of sexual harassment, a situation that limits our understanding of the experiences of nurse victims of WSH. Therefore, by exploring the lived sexual harassment experiences of nurses, we contribute to providing in-depth data to aid our understanding of the phenomenon in Ghana. This could aid in the design and implementation of targeted measures that address the issue of WSH and minimize its negative impact on nurses and increase the provision of quality healthcare in Ghana.

## Methods

This qualitative study forms part of a large ongoing research on sexual harassment in the healthcare sector in the Central Region of Ghana. The study used interpretive phenomenological approach (IPA) to explore the lived sexual harassment experiences of nurses in some selected healthcare facilities to obtain a rich understanding of the phenomenon. Interpretive phenomenology, often called hermeneutics, is underpinned by the assumptions that individual’s lived experiences can be interpreted within the context of the person’s “lifeworld” [47]. The approach focuses on describing, understanding, and interpreting human experiences [48], highlighting the embedded common life issues often taken for granted [49, 50]. Since most people in Ghana, including nurses, shy away from issues of sexual harassment, the use of IPA was deemed appropriate in the present study as it sought to explore, describe, understand and interpret the hidden sexual harassment experiences of nurses and highlight the phenomenon.

Participants are nurses (nurse assistants, registered general nurses and registered midwives) who belong to the two main nursing associations in Ghana, the Ghana Registered Nurses and Midwives Association (GRNMA) and the Union of Professional Nurses and Midwives Ghana (UPNMG) [28]. Participants were recruited after the study had been advertised on social media (WhatsApp group platforms of the GRNMA and UPNMG) using a google form survey. Interested participants were asked to leave their demographic data including their phone numbers on the google form so they can be contacted for the study. To be included in the study, participant must have worked for at least 12 months prior to data collection, should have experienced sexual harassment in the line of duty during the period, and must accept to be interviewed or provide detailed description of sexual harassment experienced via writing (using google form). All nurses who did not meet these criteria were excluded from the study. Forty-six participants responded to the study advertisement and were sent the study information via WhatsApp. The study information included an informed consent form and a google form containing the interview questions. Participants were asked to either consent to an interview or provide a written descriptive narrative of their sexual harassment experiences using the google form. Although we intended to collect data via interviews only, we made provision for written narratives to enhance participation [51], since most people in Ghana shy away from discussing sexual harassment directly. Although 33 participants provided written descriptive narratives of their sexual harassment experiences, only 13 reports were deemed clear and detailed enough and thus, were included in the final data analysis. Thirteen participants agreed to be interviewed and were called on phone by the first author and interview was scheduled. Interviews were conducted using a semi-structured interview guide via phone by the first author in the presence of the second author (See Table 1). The guide was developed by all three authors based on reviewed literature and the purpose of the study. The guide was reviewed by two experts in qualitative studies for criterion validity. Each interview session lasted 30–40 minutes and were audio recorded at the consent of the participants. Of the 13 participants who consented to be interviewed, 11 were interviewed because it was felt that no new information was being generated after the eleventh interview. Telephone interview was preferred to face-to-face due to the raging COVID-19 pandemic in Ghana at the time of data collection (24th August 2021 to 31st December 2021), and sensitive nature of sexual harassment issues.

**Table 1:** Sample Interview Guide Questions

1. Can you tell me your experiences regarding sexual harassment while working as a nurse?
2. How did you respond when you were sexual harassed?
3. How did your experience with sexual harassment at the workplace affect your health and attitude towards work?
4. What can be done to minimise or prevent the occurrence of sexual harassment at your place?

### Data analysis

Data from 24 nurses, 13 written descriptive narratives and 11 interviews were obtained. The written descriptive narratives were printed, and all interviews transcribed and printed by the lead author. The transcribed data was analyzed and coded by the lead author. The second and third author verified the data analysis process by listening to the voice recordings and reviewing the transcribed notes, assess coding and themes generated. The data analysis process followed the seven-steps approach of the IPA [29];

Step 1: The lead author read each transcribed and printed data repeatedly to ensure that he is fully immersed into the data.

Step 2: Each transcript was carefully read by the lead author, taking note of the general ideas emerging from each sentence and its association with other sentences. The noted ideas and associations were jotted in the margins of each transcript.

Step 3: Emergent themes were identified in each individual text for each participant.

Step 4: Text for emergent themes were read repeatedly together by the lead author to search for connections or determine relation with other themes.

Step 5: Related themes were compared and interpreted without compromising the individuality of each participant’s experience.

Step 6: The original interview recordings and participants written narratives were revisited frequently to ensure that generated themes reflect participants experiences.

Step 7: A final analysis of the data was done to ensure that the data is well understood by using excepts from participants own words to reflect their experiences.

### Trustworthiness

Four features of trustworthiness; credibility, transferability, dependability, and confirmability are often used in qualitative studies to enhance the reliability of the findings and replicability of the study [30]. To enhance credibility, first, the bulletin for recruiting participants for data collection was shared on the platforms of the nursing groups in the Central Region to ensure that every nurse willing to share their WSH experiences could do so. This also ensured that participants from various backgrounds got the opportunity to participate in the study and provided in-depth data. Second, the researchers ensured member checking by referring back to the participants interviewed to clarify and corroborate some of the extracted statements. For participants with written narratives, only those who clearly described their sexual harassment experiences, devoid of ambiguities, were included in the final analysis. We ensured transferability by providing the data to two non-participants sample and solicited their comments and general conceptualization of the study based on their own experiences. Dependability was ensured by engaging two experts in qualitative studies to examine the research processes for appropriateness. Finally, two other researchers with expertise in qualitative studies checked the data collection and coding processes for clarity and accuracy.

### Reflexivity

Researchers’ appreciation of the influence of their own experience and background knowledge of a phenomenon understudy is essential in ensuring rigour and phenomenological validity in qualitative research [52, 53]. Being a practising nurse and an occupational health and safety promotion researcher, the first author was interested in knowing the occurrences and the potential implications of sexual harassment on the health and safety of nurses. The second author is an expert in occupational and environmental health promotion with experience in qualitative studies, while the third author is an organisational and sports manager with rich experience in sexual harassment research in Ghana. Throughout the data analysis process, we individually kept a reflective journal to record our interpretations of emergent themes vis-à-vis the available data transcripts. Emergent themes and interpretations were discussed and critiqued by all three authors, and accepted only by consensus [53].

### Ethical consideration

This study was approved by the Institutional Review Board of the University of Cape Coast [UCCIRB], Cape Coast (UCCIRB/CES/2021/55), Ghana Health Service Ethics Review Committee [GHS-ERC], Accra (GHS-ERC 019/05/21) and Central Regional Directorate of Health Services [CRDHS], Cape Coast (CR/G-263/332). Permission was also obtained from the heads of the various nursing associations before the circulation of online survey via their WhatsApp group flatforms. We attached informed consent form to the online survey which provided the purpose of the study, eligibility criteria, anonymity, voluntary participation and confidentiality of information of participants. Consent was implied for all participants who checked “I agree” box on the google form and subsequently participated in the study.

## Results

The demographic characteristics of participants is shown in Table 2.

**Table 2:** Participant characteristics (n = 24)

Demographics	Interviews	Written Narrative	Frequency	Percentage
**Age Range**				
20–24	2	4	6	25.0
25–29	5	4	9	37.5
30–34	3	3	6	25.0
35–39	1	2	3	12.5
**Gender**				
Male	0	3	3	12.5
Female	11	10	21	87.5
**Marital Status**				
Single	7	6	13	54.2
Married	2	4	6	25.0
Living with a partner	2	3	5	20.8
**Work Experience**				
1–2 years	2	4	6	25.0
3–4 years	4	4	8	33.3
5–6 years	2	1	3	12.5
7–8 years	1	3	4	16.7
9–10 years	2	1	3	12.5
**Professional category**				
Registered General Nurse	5	6	11	45.8
Registered Midwife	3	2	5	20.8
Registered Community Nurse	0	2	2	8.3
Registered Mental Nurse	0	1	1	4.2
Enrolled Nurse/Nurse Assistant (Clinical)	2	2	4	16.7
Community Health Nurse/Assistant (Preventive)	1	0	1	4.2
**Type of Health Facility**				
Teaching Hospital	2	1	3	12.5
Regional Hospital	0	2	2	8.3
District/Primary Hospital	5	2	7	29.2
Polyclinic	1	3	4	16.7
Health Centre	3	3	6	25.0
CHPS Compound	0	2	2	8.3

Seven major themes emerged from the analysis (See Table 3); occurrence of sexual harassment, attitude towards WSH, failure to report, health impact, dealing with sexual harassment, perceived management’s attitude towards WSH and non-availability of sexual harassment preventive measures.

**Table 3:** Main themes and sub-themes extracted from the study

Focus of Study	Themes	Sub-themes
Workplace sexual harassment experience of nurses	Occurrence of sexual harassment	Verbal sexual harassment
		Non-verbal sexual harassment
		Physical sexual harassment
	Attitude towards WSH	Initial tolerance
		Late effort
	Failure to report	Fear of victimisation
		Lack of knowledge on reporting procedure
		Dissatisfaction with handling of reported cases
	Health impact	Physical health symptoms
		Psychological health symptoms
	Dealing with sexual harassment	Normalising acts of sexual harassment
		Changing of facility and attrition
	Perceived management’s attitude towards WSH	Managements’ indifference towards WSH
		Mistrust of management
	Non-availability of sexual harassment preventive measures	Lack of sexual harassment policy
		Lack of sexual harassment training

## Occurrence of sexual harassment

This theme highlighted participants’ experiences with various forms of sexual harassment in the line of duty. Three sub-themes were identified; verbal, non-verbal and physical sexual harassment.

### Verbal sexual harassment

Participants described various forms of sexually suggestive comments targeted at them while at work. These gender-based comments, often made by male perpetrators and targeted at female nurses, are used to belittle female nurses based on their sexual features or appearances. The following narrative reflect the experiences of most participants.

“*The first time I met this new doctor in his consulting room, he told me I have nice breasts, but I didn’t mind him…. He started asking about the size of my breasts…*” (Participant 3 interview: female, 27, 3 years’ experience).

### Non-verbal sexual harassment

Participants also reported their experiences of non-verbal sexual harassment such as distribution of sexually explicit materials including pictures and text. For example;

“*A patient on the ward took my contact.…then, she started sending me her naked pictures.*” (Participant 7 narrative: male, 34, 7 years’ experience).

### Physical sexual harassment

Most participants recounted various acts of physical sexual harassment including touching and fondling of victims’ sexual features. These acts were often perpetrated at various settings within the healthcare facilities including the consulting room and nurses’ room. A narrative;

“*The first time I met this new doctor in his consulting room.…Before I realised, he was already holding my breast and telling me he is measuring it.…. anytime I go to his consulting room, he’ll attempt to touch my breast and other parts of my body.*” (Participant 3 interview: female, 27, 3 years’ experience).

A male victim reported;

“*…On my night shift she chased me in the nurses’ room kissing me and doing all kinds of things.…but I managed to stop her.*” (Participant 7 report: male, 34, 7 years’ experience).

From the narratives, the perpetrators of WSH against the nurses were mostly physicians, nurses, and patients. The harassing behaviours were repetitive, perhaps due to the close and continuous working relationship between victims and the perpetrators. It is worthy to note that though these sub-themes generally reflect different forms of sexual harassment, they are not mutually exclusive because they often occur in the act of sexual harassment.

## Attitude towards sexual harassment

Initial reactions of sexual harassment victims could either deter perpetrators or allow the act to fester. Most victims reacted passively or tried to ignore the harassers at the beginning of the act, and subsequently implored on harassers to stop the harassing behaviour. However, harassers continued with the act in most cases. This theme resulted in two sub-themes; initial tolerance and late effort.

### Initial tolerance

Participants were tolerant to acts of sexual harassment when it occurred the first time. They were indifferent and did not employ on harassers to stop.

“*…but I didn’t mind him…. I held his hand and told him to stop because I don’t like what he is doing. … anytime I go to his consulting room, he’ll attempt to touch my breast and other parts of my body.*” (Participant 3 interview: female, 27, 3 years’ experience).

### Late effort

Victims only attempted to stop harassers after several harassment episodes. However, this late effort often did not stop the harassers.

“*…he always tries to touch my private parts. I gathered courage one day and told him to stop following me as well as touching me, but this man didn’t mind me and he is still doing it.*” (Participant 1 report: female, 25, 1 year experience).

“*I was thinking I can deal with it myself, but it kept happening.*” (Participant 2 interview: female, 28, 2 years’ experience).

The tolerance exhibited by most victims could embolden harassers and enhance the perpetration of harassment behaviour. Once tolerated over a period, harassers may trivialise victims’ efforts to stop them. This could result in further acts of harassment, making sexual harassment endemic in the healthcare institutions.

## Failure to report sexual harassment

Aside accommodating the act of sexual harassment, most victims did not make formal complaints or report their harassers. Failure to report acts of sexual harassment to the appropriate authorities was associated with three sub-themes.

### Fear of victimisation

Most participants could not report their sexual harassment ordeal due to the fear of being victimised by harassers especially when the harasser is a person in a position of seniority or authority.

“*Because his wife was also a senior nurse in the same hospital, I didn’t want to create problem for myself as a junior staff, so I couldn’t report him.*” (Participant 11 report: female, 24, 1 years’ experience).

“*Hmmm…How can I report a whole director? I’m afraid, I don’t know what will happen to me when I report him.*” (Participant 6 interview: female, 33, 7 years’ experience).

### Lack of knowledge on reporting procedure

Some of the participants suggested they did not know who to report to or how to file a formal complaint. This is exemplified in the following narrative.

“*Honestly, I wanted to report him, but I just didn’t know who to report to. I felt I must talk to somebody, but I don’t know who to trust. I kept on thinking about who should I talk to…. definitely, not my in-charge.*” (Participant 2 interview: female, 28, 2 years’ experience).

### Dissatisfaction with handling of reported cases

Participants who were dissatisfied with handling of reported cases by their management were reluctant to report harassers. They did not also have much confidence in reporting to their immediate supervisors. Example;

“*I wish I could have reported it to my unit head, but I know she will not take action because some of my colleagues told me they have reported the guy* [physician] *before and she did not do anything. I also don’t want to go straight to the nurse manager or administrator.*” (Participant 13 report: female, 29, 5 years’ experience).

Reporting work-related sexual harassment is essential in highlighting the scope of the problem which could influence intervention and policy formulation. Thus, lack of reporting limit our appreciation of the phenomenon and encourages perpetration of the act.

## Health impact of sexual harassment on victims

Most of the participants reported physical and psychological symptoms which they attributed to the act of sexual harassment. These physical and psychological symptoms often occurred together in one episode of sexual harassment.

Physical health impact.

Some participants reported symptoms of physical ailments such as headache and palpitations. Example;

“*…and by morning time, I’ll be having severe headache… and get palpitations….*” (Participant 1 interview: female, 27, 2 years’ experience).

Psychological health impact.

Participants also reported symptoms of psychological disorders including fear, anxiety, and PTSD as typified in the following narrative;

“*…because he attempted to sleep with me in the nurses’ room, I’m always scarred when I’m alone in the room. I always feel like someone will come and pounce on me when I enter the room…. anytime I’m going to work, I become anxious…. At times I can’t sleep at night….*” (Participant 1 interview: female, 27, 2 years’ experience).

## Dealing with sexual harassment

While some participants dealt with sexual harassment by normalising the behaviour, others changed facility or intend to change or leave the nursing profession just to avoid harassers.

## Normalising the acts of sexual harassment

As a way to cope with the harassment, some participants accepted that sexual harassment is part of nursing work, but that is almost a hopeless situation. An interviewee narrated:

“*…I used to be offended any time he touched me. But now I don’t worry too much, I’m not comfortable with it but…. After all, I don’t know how to stop him. …again, and I can’t also say I’ll not work with him. So, what can I do? My friend would say it is part of the job [*laughter*].*” (Participant 9 Interview: female, 29, 5 years’ experience).

## Changing facility and attrition due to sexual harassment

Some participants who were unable to deal with the harassment changed their facilities, by taking a transfer to another facility to avoid the harasser. This often happened when the perpetrator is a senior colleague or a person in a position of power. A victim “cried”:

“*I was sexually harassed at my place of work by a management member on many occasions…. I decided to take a transfer to another facility because I couldn’t bear it anymore.*” (Participant 5 report: female, 26, 2 years’ experience).

Others were eager to change their facility or quit their job just to avoid the harassers. For instance:

“*There was this male nurse who proposed love to me and I refused. Because he is a senior to me at the workplace, he always wants to embarrass me and bully me. It makes me very uncomfortable any time I’ve to work with him. I wish I could even get a transfer to another facility because of him.*” (Participant 10 report: female, 27, 2 years’ experience).

“*There is this hospital administrator who keep demanding sexual favours from me. I tried all I can to stop him but he will not. Now I don’t even know what to do again. Because of him I have applied for transfer but I’m still not getting it. I wish I could just resign and leave this hospital.*” (Participant 4 report: nurse, female, 31, 4 years’ experience).

Participants’ handling of sexual harassment episodes could generally be described as ineffective and inimical to the fight against WSH. Acts of normalisation, changing of facility and attrition only exposes more nurses to sexual harassment and do not stop harassers from perpetrating the act.

## Perceived management’s attitude towards sexual harassment

Most participants suggested that management pay no attention to sexual harassment issues at the workplace.

Managements’ indifference towards WSH.

Managers of the healthcare institutions were generally seen as being indifferent or unconcerned regarding issues of sexual harassment, especially on handling of reported cases. An interviewee lamented:

“*I don’t think management is doing anything to stop the sexual harassment. Because the guy who harassed me, I hear he has done it to many staff and even students and rotation nurses. One of my colleagues reported him and they did nothing to him and he’s still doing it. Management is not doing anything to stop him. For now, it looks like we are on our own.*” (Participant 4 interview: nurse, female, 35, 10 years’ experience).

Mistrust of management.

Some participants did not trust management will effectively handle their cases and thus, were unwilling to report sexual harassment ordeals. Example;

“*…it is also difficult to trust management and share my problem with them.*” (Participant 4 report: nurse, female, 31, 4 years’ experience).

## Non-availability of sexual harassment prevention measures

Availability of sexual harassment prevention measures are essential in addressing the phenomenon at workplaces. Most of the participants indicated limited awareness of sexual harassment preventive measures including policies and training at the workplace.

### Lack of sexual harassment policy

Most participants indicated that they have never seen or heard of a sexual harassment policy at their workplaces. A participant reported.

“*No, we don’t have anything like that* [sexual harassment policy]. *I’ve been working like 7 years now, but I’ve never seen anything like sexual harassment policy at my facility. I’ve never heard it before. Even if GHS has it, for here we don’t, we don’t.*” (Participant 6 interview: female, 33, 7 years’ experience).

However, a few participants mentioned some of the existing workplace policies such as the code of conduct and disciplinary procedures of the GHS as policies used to address issues of sexual harassment at the workplace.

“*I’ve seen sexual harassment in the GHS code of ethics book. So, they* [perpetrators of sexual harassment] *should know that it is an offense and stop harassing people…. I think that is what management will use if someone should report to them. But I didn’t have the confidence to report mine, so can’t tell but I think they will use that.*” (Participant 8 interview: nurse, female, 30, 5 years’ experience).

### Lack of sexual harassment training

None of the participants had ever received training on sexual harassment since joining the nursing profession. As expressed by a participant.

“*No, not at all, I’ve never received anything like sexual harassment training.*” (Participant 6 interview: nurse, female, 33, 7 years’ experience).

The importance of policies and training in prevention of sexual harassment at the workplace cannot be overemphasised. Aside increasing occurrence of sexual harassment at the workplace, the non-availability of these measures could negatively affect resolution of cases and increase the negative physical and psychological health outcomes among the victims, and compromise delivery of quality healthcare.

## Discussion

This study used data from participants’ written descriptive narratives and interviews to explore the lived WSH experiences among nurses in healthcare facilities in the Central Region of Ghana. Our findings revealed that participants experienced verbal, non-verbal, and physical forms of sexual harassment, often perpetrated by physicians, other workers, and patients. We also found that most victims reacted passively to sexual harassment behaviours and cases were often not reported despite the negative impact on victims’ health. Furthermore, our findings revealed that while some participants coped by accepting sexual harassment as an inevitable part of their job, others either quit or intended to quit their job to avoid the harassers. Besides, we found that most participants were unaware of any WSH preventive measures or policies in the healthcare sector in Ghana.

Consistent with the findings from previous studies in Ghana [22] and elsewhere [16, 20, 21], this study found that nurses experienced verbal, non-verbal and physical forms of sexual harassment which occurred repeatedly and were often perpetrated by physicians, other nurses, and patients. Perhaps, the close working relationship between the perpetrators and the nurses explains the repetitiveness of the harassing behaviour in most cases. Nurses are mostly harassed by people with whom they work closely [20, 21] which could have serious negative repercussion on the working relationship between the nurse and the perpetrator and thus, affect healthcare delivery, unless it is addressed properly [31]. This emphasises the need for stringent measures against WSH at healthcare facilities.

The reactions of victims of sexual harassment could determine whether perpetrators will continue with the act of harassment or stop it. Like the findings from previous studies [14, 21], most victims in the present study reacted passively or attempted to ignore the harassers, and only implored on harassers to stop when the harassing behaviour continued. This could be attributed to the fact that most perpetrators are people in senior position to the victims [32] or victims have limited knowledge on how to handle sexual harassment issues at the workplace [19, 33]. Available evidence suggests that most perpetrators of sexual harassment against nurses tend to continue perpetrating the act because victims often react passively [15]. Thus, it is not surprising that most harassers continued with the act of harassment even after victims have attempted to stop them. Therefore, it is important to educate nurses on how to deal with perpetrators of sexual harassment, especially during the initial stages of the harassment behaviour. Such action could limit the occurrence of sexual harassment, especially the severe forms of harassment such as sexual assault and its’ attendant consequences. This could also prevent continuous perpetuation of the act of harassment and minimise its negative impact on the health and safety of the nurses.

Despite the importance of reporting in the resolution and prevention of sexual harassment cases at workplaces, most victims in the current study did not report or make formal complaint to their leaders or management. Evidence suggests that most sexual harassment victims are afraid to report because they think they could be victimised by the same superiors who harassed them or even their colleagues [14, 34]. Thus, as found in the current study, most victims alluded to fear of victimisation as one of the major reasons for not reporting, and that most of the harassers are in position of power [35, 36]. Additionally, the burden of proof associated with reporting cases of sexual harassment often deter many victims from making formal complaints [24] which encourage perpetuation of the act.

Our findings also revealed that while most victims did not know who to report to or how to file a formal complaint, others did not have confidence in reporting to their immediate supervisors or in-charges. Similar findings were reported in a previous study [37]. The nonavailability of sexual harassment reporting mechanisms at most workplaces remains a major hinderance towards reporting of WSH cases [37]. Victims of sexual harassment often fail to report harassers to the appropriate authorities because they often do not trust the institutional processes that deal with reported cases [24]. These findings emphasise the need for evidence-based sexual harassment reporting protocols or procedures that encourage victims to report cases while protecting them from any reporting-related repercussions. Perhaps, providing explicit and multiple sexual harassment reporting avenues that ensure confidentiality, and permit victims to bypass their organisations when filling complaints, could encourage reporting of sexual harassment cases and ensure effective resolution of such cases.

We also found that, victims experienced various physical and psychological health symptoms including headache, palpitation, sleeplessness, anxiety, and PTSD. This finding is in concordance with that from previous studies [33, 38]. The health-related consequences of WSH often contribute to absenteeism, presenteeism, and job withdrawal which affect quality healthcare delivery, patient safety, and health outcomes [39, 40]. Besides, the negative health impacts of sexual harassment are exacerbated in organisations where victims perceive management to be highly tolerant to issues of sexual harassment [39], and victims’ perception about the likelihood of harassment recurring is high [41]. Therefore, it is important for managers of the healthcare facilities to take sexual harassment serious and address victims’ grievances. This could minimise the negative health implications of sexual harassment on victims and its associated consequences on healthcare delivery.

Like the findings from previous studies [19, 42], we found that some participants tried to cope with the acts of sexual harassment by accepting the behaviour as an inevitable part of their work as nurses. Such attitude towards the acts of WSH may be attributed to victims’ attempt to minimise the psychological impact of sexual harassment on their life and work delivery. This perceived normalisation of sexual harassment at most workplaces [33] had been implicated as one of the main reasons sexual harassment has become endemic in the healthcare settings, especially against nurses [19, 42]. We further found that victims who were unable to cope with acts of sexual harassment at their workplaces either moved to different healthcare facilities or had the intent to move to avoid the harassers. Similar findings were reported in previous studies [24]. Regrettably, some victims expressed the intention to quit their job if such harassment acts continue and they are unable to change facility. This often happens when the harasser is a person in position of power or authority [43]. This could negatively affect nursing human resource management and contribute to nurse shortage, thereby reducing quality of healthcare delivery [42–44].

We also found that management of healthcare facilities (in this study) pay no or little attention to sexual harassment issues at these healthcare facilities. This could promote tolerance and increase the occurrence of sexual harassment [3, 39], thereby compromising the health and safety of the nurses. Meanwhile, evidence suggests that managements’ approach to handling sexual harassment grievances often determines whether victims will quit their jobs or will stay, and whether they will seek legal remedy or not [11]. Perhaps, increasing sexual harassment awareness among nurses, and training management in sexual harassment grievance handling would minimise occurrences and the negative consequences of sexual harassment in the healthcare sector.

Our findings also revealed that most of the participants in this study were not aware of sexual harassment preventive measures at their facilities, including the availability of sexual harassment policy. This could be due to the nonavailability of a documented policy on sexual harassment within the care sector in Ghana, which makes it difficult to address the phenomenon [25]. Although, a few of the participants identified some of the existing workplace policies such as the Code of Conduct and Disciplinary procedures of the GHS [45] and the Occupational Health and Safety Policy Guidelines for the health sector [46], these policies do not explicitly address sexual harassment in the healthcare sector. Thus, to effectively address sexual harassment among nurses, there is the need for a policy document that explicitly outlines measures targeted at addressing the issue of sexual harassment within the healthcare sector. Additionally, providing sexual harassment training to workers in the healthcare sector could enlighten both victims and perpetrators towards the act and therefore, enhance the implementation of anti-sexual harassment policies, to reduce or stop the act.

## Strengths and limitations

This study provides a deep insight into the lived WSH experiences among nurses working at the healthcare facilities in the Central Region in Ghana. Thus, the study contributes to our understanding of the phenomenon which remains largely unexplored from a qualitative perspective among nurses in Ghana. The circulation of the data collection bulletin across various WhatsApp platforms for nurses ensured that professionals with different qualifications and from varied healthcare facilities participated in the study. Despite these strengths, there are some limitations that need our attention. First, the experiences of sexual harassment among the nurses were self-reported. This could introduce social desirability bias, especially, because of the sensitivity and perceived embarrassment associated with sexual harassment within the Ghanaian cultural settings. Perhaps, the use of online and telephone data collection strategies reduces such social desirability and improve the trustworthiness of our findings. Second, because the survey was circulated via online platforms for nurses, only those with smartphones and have access to internet during the period of data collection could participate in this study. Also, despite targeting both male and female nurses, only a few male nurses were involved in the study. Thus, it was difficult to appreciate the lived experiences of male nurses with WSH.

## Recommendations

Considering the impact of WSH on the health and safety of nurses as well as quality healthcare delivery, it is important for healthcare managers and other stakeholders to prioritise the issue WSH in healthcare and implement strategies to prevent its occurrence. For instance, managers of healthcare institutions can demonstrate high level of intolerance to acts of WSH through position statement that unequivocally abhors the behaviour in the healthcare facilities. Also, developing a comprehensive anti-sexual harassment policy and making it available to all staff, providing complaint procedure and grievance handling processes that could bypass victims’ direct supervisors, and regular training of healthcare workers on WSH might minimise the risk of sexual harassment occurring in the healthcare sector, especially against nurses. Also, due to the limited data on WSH among male nurses, future studies could target only male nurse victims of sexual harassment in order to aid our understanding of the phenomenon from their perspectives.

## Conclusion

Findings from this study highlights the problems of sexual harassment against nurses in the healthcare sector in Central Region of Ghana, which is rarely acknowledged, especially from the perspectives of victims. Our findings revealed that the nurses experience verbal, non-verbal and physical form of sexual harassment, often perpetrated by physicians, other workers, and patients. Most victims also react passively to sexual harassment behaviours and cases are often not reported despite the negative impact on victims’ health. While some participants coped by accepting sexual harassment as an inevitable part of their job, others either quit or intend to quit the job to avoid the harassers. Most participants are unaware of any workplace policy or strategy that addresses sexual harassment in the sector. These findings could aid the various stakeholders and policy makers in the healthcare sector in Ghana to design and implement measures such as a sexual harassment policy to prevent occurrence and effectively resolve cases when one occurs. Besides, improving sexual harassment awareness among nurses, and other workers in the healthcare sector could enlighten both victims and perpetrators on the negative repercussions of the phenomenon and thereby limit occurrences.

## Data Availability

The datasets generated and analyzed during the current study are available from the corresponding author on reasonable request.

## List of abbreviations

GHS: Ghana Health Service
GRNMA: Ghana Registered Nurses and Midwives Association
IPA: Interpretative phenomenological approach.
LMICs: Low- and middle-income countries
PTSD: Post-traumatic stress disorders
UPNMG: Union of Professional Nurses and Midwives Ghana
WSH: Workplace sexual harassment
